# Diversity of greek meningococcal serogroup B isolates and estimated coverage of the 4CMenB meningococcal vaccine

**DOI:** 10.1186/1471-2180-14-111

**Published:** 2014-04-29

**Authors:** Georgina Tzanakaki, Eva Hong, Konstatinos Kesanopoulos, Athanasia Xirogianni, Stefania Bambini, Luca Orlandi, Maurizio Comanducci, Alessandro Muzzi, Muhamed-Kheir Taha

**Affiliations:** 1 National Meningitis Reference Laboratory, National School of Public Health Athens, 196, Alexandras Avenue, Athens, Greece; 2 Institute Pasteur, Invasive Bacterial Infections Unit, Paris, France; 3 Novartis Vaccines and Diagnostics, Siena, Italy

**Keywords:** 4CMenB vaccine, MATS, Vaccine, Vaccine coverage, Meningococcal disease

## Abstract

**Background:**

Serogroup B meningococcal (MenB) isolates currently account for approximately 90% of invasive meningococcal disease (IMD) in Greece with ST-162 clonal complex predominating. The potential of a multicomponent meningococcal B vaccine (4CMenB) recently licensed in Europe was investigated in order to find whether the aforementioned vaccine will cover the MenB strains circulating in Greece. A panel of 148 serogroup B invasive meningococcal strains was characterized by multilocus sequence typing (MLST) and PorA subtyping. Vaccine components were typed by sequencing for factor H-binding protein (fHbp), Neisserial Heparin Binding Antigen (NHBA) and *Neisseria* adhesin A (NadA). Their expression was explored by Meningococcal Antigen Typing System (MATS).

**Results:**

Global strain coverage predicted by MATS was 89.2% (95% CI 63.5%-98.6%) with 44.6%, 38.5% and 6.1% of strains covered by one, two and three vaccine antigens respectively. NHBA was the antigen responsible for the highest coverage (78.4%), followed by fHbp (52.7%), PorA (8.1%) and NadA (0.7%). The coverage of the major genotypes did not differ significantly. The most prevalent MLST genotype was the ST-162 clonal complex , accounting for 44.6% of the strains in the panel and with a predicted coverage of 86.4%, mainly due to NHBA and fHbp.

**Conclusions:**

4CMenB has the potential to protect against a significant proportion of Greek invasive MenB strains.

## Background

In the global effort to eliminate bacterial meningitis and septicemia, serogroup B *Neisseria meningitidis* is among the most challenging pathogens for vaccine development [1,2]. This is due to the fact that serogroup B capsular polysaccharide is not immunogenic and is a potential self-antigen [3,4]. The approach of strain-specific outer membrane proteins has been successful in the development of vaccines effective against homologous strains [5,6].

A novel multicomponent vaccine against invasive disease caused by meningococcal capsular group B (MenB), 4CMenB (Bexsero®), containing four major components: factor H-binding protein (fHbp) [7], Neisserial Heparin Binding Antigen (NHBA) [8], *Neisseria* Adhesin A (NadA) [9], and outer membrane vesicles (OMV) derived from the New Zealand outbreak strain NZ98/254 [10], has been recently developed and licensed in Europe.

For vaccines based on meningococcal serogroups A, C, W and Y capsular polysaccharide conjugates which have been licensed in many parts of the world [11-13], the immunogenicity has been evaluated by means of complement–mediated killing using the serum bactericidal assay (SBA) of 4 strains belonging to each serogroup and the coverage is estimated on the basis of the epidemiological serogroup distribution [14-16]. This is very difficult for the evaluation of the novel recombinant protein-vaccine that aimed to target serogroup B due to the fact that the protein antigens may vary in their sequence and level of expression across strains [17]. Phase variation, gene regulation, and sequence diversity can in fact affect the quantity of the target protein antigens on the bacterial surface or the cross-reactivity of these surface proteins with those contained in the vaccine. This diversity significantly impacts the likelihood that vaccine-induced antibody responses will kill any given MenB isolate. This variability across strains would thus require extensive testing in SBA with human complement (hSBA) when assessing large strain panels. Such testing is clearly problematic because of the difficulty to standardize the hSBA across diverse strains and sources of human complement. For this reason, alternative means of measuring the probability of killing in the hSBA by antibodies induced by the surface protein based vaccine are necessary [18].

The Meningococcal Antigen Typing System (MATS) is an ELISA developed to evaluate whether a given strain expresses at least one of the antigens (fHbp, NHBA and NadA) contained in the 4CMenB vaccine and the degree of cross-reactivity [19]. MATS also considers the PorA variable region 2 (VR2) of the target bacteria in order to assess the immunodominant contribution of the outer membrane vesicle (OMV-NZ) from the New Zealand outbreak strain, which possesses PorA P1.4, to the 4CMenB vaccine [20]. Strains that meet a minimum threshold of reactivity to fHbp, NadA or NHBA in the MATS ELISA, named positive bactericidal threshold (MATS-PBT), or that possess the PorA VR2 4 are expected to be covered by 4CMenB [19]. The baseline relationships of MATS to hSBA represented by the MATS-PBT values were established using pooled sera obtained from infants following a three dose primary series of 4CMenB vaccine and a booster dose at 12 months of age. The MATS ELISA was then transferred to several National Meningococcal Reference Laboratories and an interlaboratory standardization study was conducted to ensure consistent results across European reference laboratories that allowed testing the strain coverage in Europe and Canada [21-24].

Although the incidence of the Invasive Meningococcal Disease (IMD) in Greece decreased from 1.94 in 1999 to 0.68 per 100,000 in 2009 upon the introduction of conjugate MenC vaccine [25], serogroup B currently accounts for approximately 90% of the IMD similarly to other European countries. The predominant clonal complex (cc), cc162, is proportionally higher as compared to other European countries, where it represents only 2.5% of invasive isolates, as recently published in a study conducted in five European countries (Euro-5) [23]. The aim of the present study was to investigate the potential coverage of 4CMenB meningococcal vaccine in Greece, with particular regards on the impact that the cc162 has on this coverage.

## Methods

### Meningococcal isolates, PCR and sequencing

**Figure 1:**
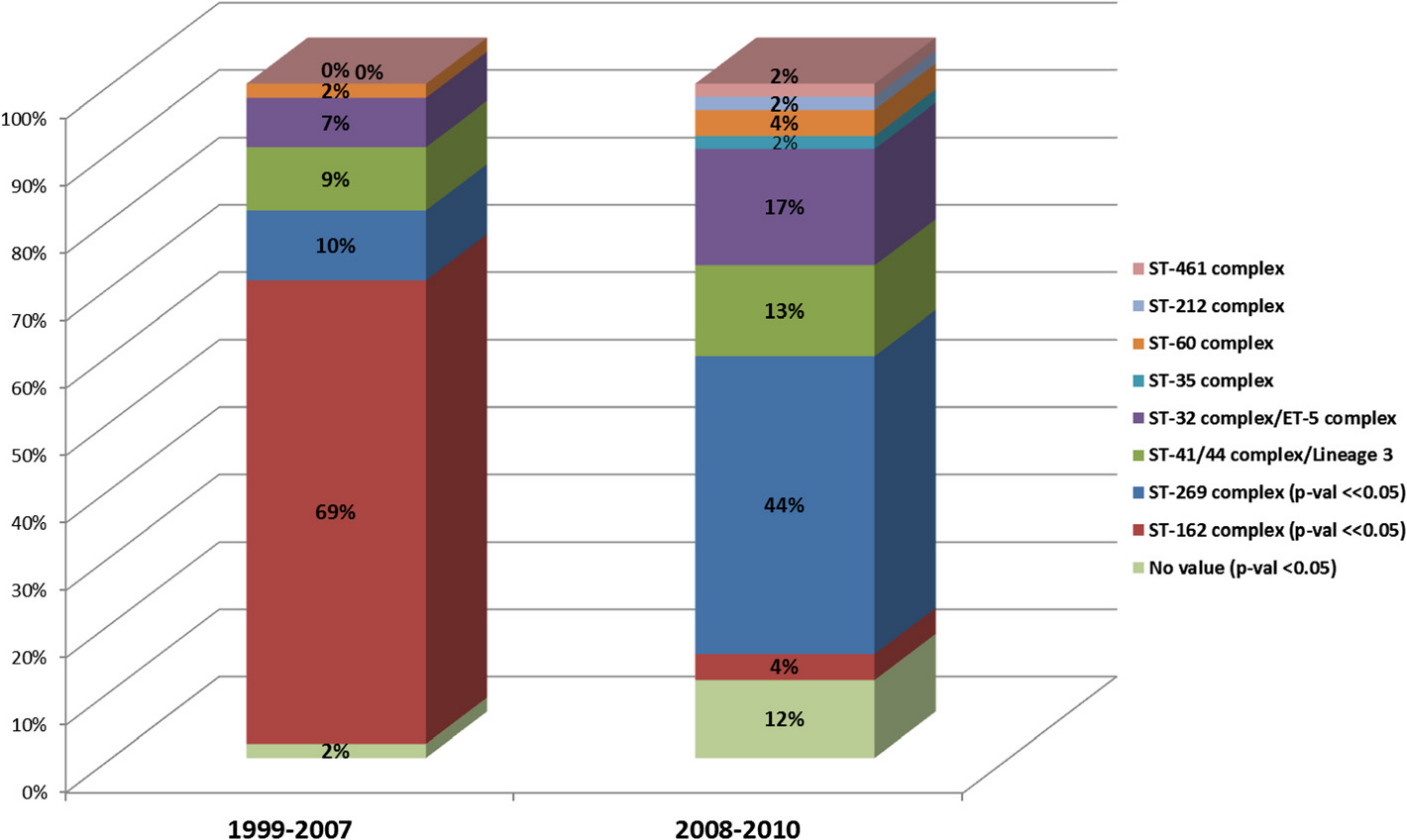
Most frequent clonal complexes among the two subsets of 96 (1999–2007) and 52 (2008–2010) Greek isolates.

A total of 148 serogroup B meningococcal strains isolated from cases of IMD during an 11 year period (1999–2010) collected -as part of standard patient care- by the National Meningitis Reference Laboratory (NMRL) at the National School of Public Health in Athens, Greece, were studied retrospectively. This strain set is composed of: a first subset of 52 clinical revived isolates out of the 58 (90%) collected by the NMRL during 2008–2010, representative of endemic MenB disease burden in Greece during that period; a remaining subset of 96 strains isolated from 1999 to 2007, specifically enriched for the cc162 (n = 66 in this subset), which was highly prevalent in Greece but is decreasing in recent years, and for the cc269 (n = 10 in this subset), which has recently emerged in Greece (Figure 1). All strains were PorA subtyped using both serosubtyping and genosubtyping, by sequencing of the three Variable Regions VR1, VR2 and VR3 of the *porA* gene [26-29]. The deduced amino acid sequences of VR1 and VR2 were assigned according to the *Neisseria meningitidis* PorA Variable Regions Database (http://neisseria.org/nm/typing/pora). The PorA VR3 database (http://www.shlmprl.scot.nhs.uk/PorA_VR3.asp) was used to assign VR3 subtypes.

Strains were characterized by MLST following the guidelines included in the public MLST database (http://pubmlst.org/neisseria/); PorA, NHBA and NadA sequence variants (alleles and peptides) have been assigned using the same interface as MLST. PCR and gene sequencing of *fHbp* and *nhba and nadA* gene presence were evaluated by previously published methods [9,30-33]. The new alleles were deposited in GenBank under the accession numbers KJ567159 to KJ567306 and KJ567307 to KJ567449 for the *fHbp* and *nhba* respectively. Assembly of the sequences was performed using the Sequencer program version 4.10.1 (Gene Codes Corporation) and Vector NTI suite v11. Sequences were aligned by BioEdit http://www.mbio.ncsu.edu/BioEdit/bioedit.html.

### MATS

All isolates were analyzed by MATS ELISA to determine the proportion of strains expected to be covered by 4CMenB. MATS ELISA was carried out at Meningococcal Reference Laboratory, the Institut Pasteur (Paris, France) which is one of the MATS reference laboratories which participated to the inter laboratory standardization [22,23]. MATS ELISA values were calculated as antigen-specific relative potencies compared with MenB reference strains expressing each vaccine antigen [19,22]. The data were compiled and quality controlled by Novartis Vaccines and Diagnostics.

### MATS-PBT prediction of 4CMenB strain coverage

Predicted coverage using MATS-PBT was calculated as described previously [19,22,23]. The presence of at least one antigen with a relative potency greater than its MATS-PBT relative potency value (0.021 for fHbp, 0.294 for NHBA and 0.009 for NadA) or the presence of PorA VR2 1.4 (matched to the OMV-NZ component of 4CMenB) was considered to be sufficient for a strain to be covered by 4CMenB. Strains that did not meet these criteria were considered not covered. Estimates of the 95% confidence intervals (95% CI) for the MATS-PBTs were derived on the basis of overall assay repeatability and reproducibility (0.014-0.031 for fHbp, 0.169-0.511 for NHBA, 0.004-0.019 for NadA) [22]. These intervals were used to define the 95% strain coverage interval by 4CMenB.

## Results and discussion

### Prevalence and diversity of the tested isolates

**Figure 2:**
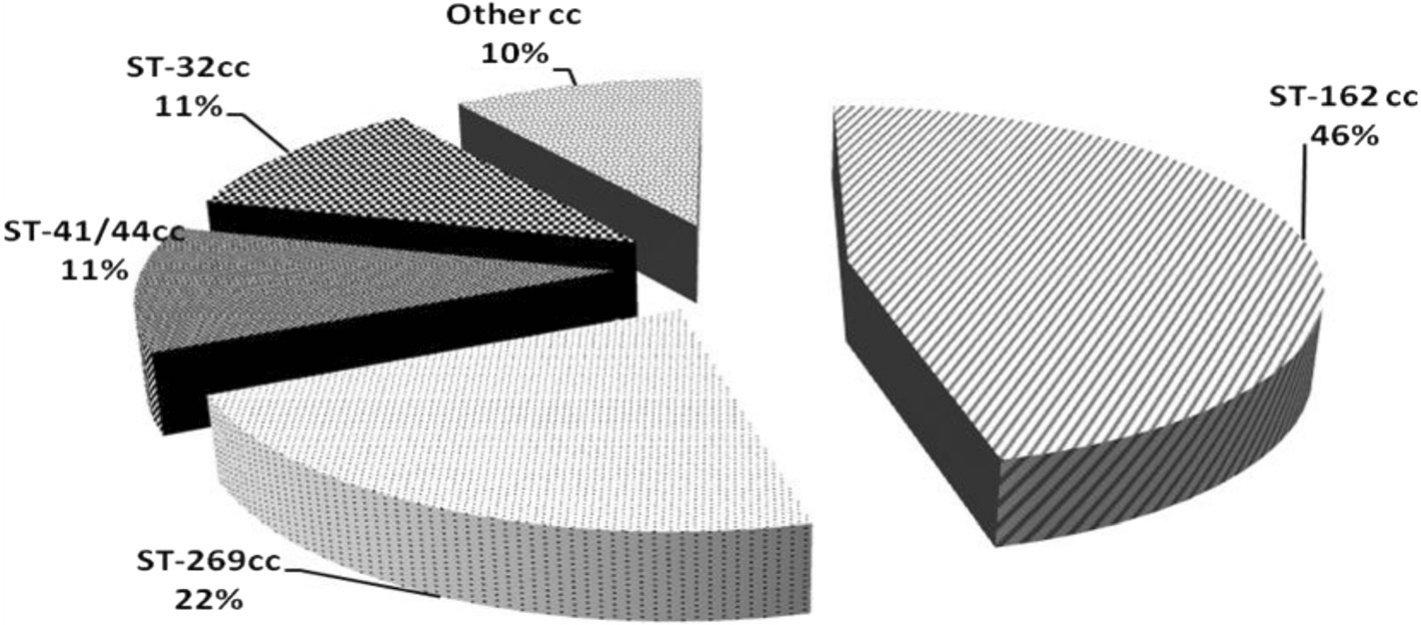
**Most frequent clonal complexes among the 148 Greek isolates (1999–2010).** The percentages of isolates within each clonal complex that were covered by at least the indicated protein are displayed.

The tested isolates belonged to several clonal complexes (cc). Among the 148 isolates tested by MATS, 66 (44.6%) belonged to cc162, which is the predominant lineage in Greece, followed by cc269 (33/148; 22.3%), cc41/44 (n = 11/46; 24%) and cc32 (18/148; 12.1%) each respectively, while 15 isolates (15/148; 10.1%) belonged to other clonal complexes (cc) (cc60, cc35, cc461, cc212) or to sequence types (STs) not currently assigned to any clonal complex (Figure 2). The proportion of clonal complexes in Greece was different as compared with other European Countries, based on data recently published by Vogel and colleagues in the Euro-5 study [23] this was particularly true in the case of cc162, which was 44.6% in Greece but which represented only 2.5% in other European Countries, at least based on combined data from Germany, France, Italy, United Kingdom and Norway and on preliminary data from Spain and Czech Republic. The percentage of isolates belonging to cc269 was 22.3% in Greece, higher than in the rest of Europe, however it was quite comparable with data from United Kingdom. On the contrary, the proportion of cc41/44 isolates in Greece, 12.1% was slightly lower with respect to other European Countries.

Greek isolates, including those belonging to the same clonal complex, showed several combinations of variable regions 1 and 2 (VR1 and VR2) in PorA. The OMV component of the vaccine contains PorA subtype P1.7-2, 4. 11 isolates among the 148 analysed (7%) showed this subtype. However, the immune response induced by PorA has been shown to specifically target the VR2 4 epitope [34]. Twelve (12) isolates (8.1%) showed VR2 4, with nine of them belonging to cc41/44. The percentage of PorA VR2 4 in the other European Countries was about 20%, higher than in Greece.

The most common fHbp variant in Greece was variant 1 (66.9%) followed by variant 2 (24.3%) and variant 3 (8.8%). Among the fHbp peptides the most common was 1.15 (41/148, 27.7%) followed by peptide 2.21 (25/148, 16.9%) and 1.1, corresponding to the specific genotype included in the 4CMenB vaccine (16/148, 10.8%). This differed from the EURO-5 study, in which peptide 1.4 (16.2%) was the most frequent and peptides 1.15 and 2.21 were identified only in 11.4% and 2% of isolates, respectively, whereas the percentage of fHbp-1.1 was quite comparable [23]. The NHBA peptide 20 (63/148, 42.6%), 21 (33/148, 22.3%) and 2 (15/148, 10.1%) accounted for more than 75% of the strains. This also differed from the Euro-5 study [23] where the peptide 2 was the most frequent (24.7%) and the peptide 20 was represented by 5% of the isolates. NHBA peptide 20 was predominant in Greece as a consequence of the prevalence of cc162. For NadA, 18 of 148 (12%) isolates harbored *nadA* gene (22.3% in the EURO-5 study), including one cc41/44 isolate, one cc212 isolate and all cc32 isolates. The remaining isolates were devoid of *nadA* gene. The *nadA* gene presence was slightly lower in Greece than in the rest of Europe.

### Estimated 4CMenB coverage

The analysis of Greek strains revealed that the coverage by at least one antigen (fHbp, NHBA, NadA or PorA) predicted by MATS was 89.2% (63.5%-98.6%) CI_0.95_ by at least one antigen (Table 1). This prediction is similar to the coverage predicted by MATS-PBT for only the 52 strains that were collected in Greece during 2008–2010, which was 88% (60%-96%) CI_0.95_. The predicted coverage for each of the clonal complexes is shown in Table 2. The highest predicted coverage was shown among the strains belonging to cc32/ET-5 (100%), followed by cc269 (97% (57.6%-100%) CI_0.95_), cc41/44/lineage3 (94.4% (72.2%-100%)CI_0.95_) and cc162 (86.4% (63.6%-100%) CI_0.95_).

**Table 1:** Contribution of each antigen and their combination to MATS PBT predicted coverage

**Antigen Combination**	**No of strains**	**% coverage of each antigen combination**	**% coverage of combined antigen groups**
No antigen	16	10.8%	10.8%
fHbp	14	9.5%	
NadA	1	0.7%	44.7%
NHBA	50	33.8%	
PorA	1	0.7%	
fHbp + NHBA	55	37.1%	
PorA + NHBA	2	1.3%	44.5%
PorA + fHbp + NHBA	9	6.1%	

**Table 2:** MATS-PBT predicted coverage by clonal complex

**Clonal Complex**	**No of Strains**	**Predicted coverage**
ST-162	66	86.4% (63.6%-100%)CI_0.95_
ST-269	33	97.0% (57.6%-100%)CI_0.95_
ST-41/44/lineage 3	18	94.4% (72.2%-100%) CI_0.95_
ST-32/ET-5	16	100%

**Figure 3:**
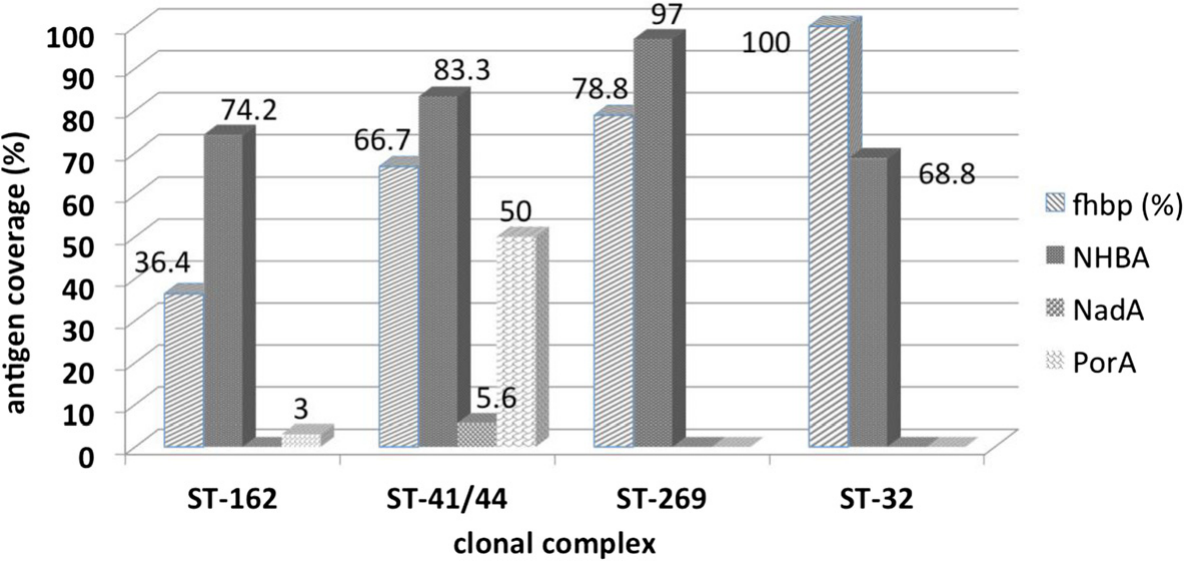
**Contribution of each antigen to coverage in relation to clonal complex.** The numbers indicate the percentage of isolates predicted to be covered by each individual antigen. Isolates were defined as covered if they expressed PorA VR2 4 or had a MATS relative potency greater than the positive bactericidal threshold (PBT) for fHbp, NHBA, or NadA.

The contribution of each antigen to coverage was variable across the clonal complexes (Figure 3). NHBA showed the highest contribution (116/148; 78.4%) followed by fHbp (78/148; 52.7%), PorA (12/148; 8.1%) and NadA (1/148; 0.7%) (Table 1). The overall contribution of NHBA was consistently high across clonal complexes presenting coverage values from 11/16 (68.8%) for cc32, 49/66 (74.2%) for cc162, 15/18 (83.3%) for cc41/44 while the highest value was found among the cc269 isolates (32/33; 97%).

The lowest fHbp contribution was found among the cc162 (24/66 36.3%) while higher contributions were found among cc41/44 (12/18; 66.7%), cc269 (26/33; 78.8%) and cc32 isolates (16/16; 100%). PorA contribution to coverage in relation to clonal complexes revealed that PorA 1.4 was found mainly among the cc41/44 (9/18; 50%) while low PorA contribution was found for cc162 (2/66; 3%) and no PorA contribution to coverage was found for cc269 and cc32 strains. In contrast, NadA contribution to coverage was low among cc41/44 isolates (1/18; 5.6%), while it was not found in other clonal complexes (Figure 3).

The recent licensure of the 4CMenB vaccine in Europe may promote recommendations for its use by national immunization technical advisory groups. Data on strain coverage are therefore crucial for decision making. This study provides the first such data on the potential coverage of Greek MenB isolates by 4CMenB. The relevance of this study is related to the high incidence, in Greece, of cc162, which is rare in Europe. cc162 has been described to be present both in disease-associated and in carrier isolates in Greece, with a high degree of heterogeneity among the isolates [35,36].

When compared with killing of MenB strains in the hSBA, MATS-PBT was shown to provide a conservative prediction of strain coverage, especially in older age groups (children, adolescents, and adults) [37]. Notably, the MATS assay was not designed to assess synergistic killing effects for strains having multiple MATS relative potencies for different antigens slightly below their positive bactericidal thresholds. Using this conservative predictor, the 4CMenB vaccine is expected to provide good strain coverage globally (89.2%) among the tested isolates (148 strains isolated from cases of IMD during 1999–2010) and in particular for the most prevalent ccs, which include cc162 and cc269 predicted to be covered at 86.4% and 97%, respectively. The components of the 4CMenB vaccine contributed to MATS-PBT predicted strain coverage singularly (for a total of 44.6% of strains covered by one antigen) or in combination each other (44.6% covered by two or more antigens). A key antigen contributing to the coverage of Greek isolates was NHBA, predicted to cover the 78.4% of isolates. The greater contribution of NHBA to coverage with respect to the other antigens was evident for three out of the four most frequent MLST genotypes in Greece, cc162, cc41/44 and cc269. It was particularly relevant in the case of cc162, for which NHBA was predicted to cover 74.2% of isolates, whereas fHbp was predicted to cover only 36.4% of isolates, due to a relative high proportion of fHbp variant 2 and 3. The sequence homogeneity of NHBA in isolates belonging to cc162, quite always containing peptide 20, and its high contribution to predicted coverage are of interest also due to the already described heterogeneity of this clonal complex in Greece. Moreover, our results suggest a strong association between NHBA peptide 20 and predicted coverage. In contrast, contribution of NadA to MATS-PBT predicted strain coverage was particularly low in Greek isolates although the encoding gene was present in 12% of isolates. However, recent data suggest that *nadA* expression is repressed under the MATS assay experimental conditions and that this repression is attenuated by 4-hydroxyphenylacetic acid, a natural molecule released in human saliva, thus leading to the de-repression of *nadA* in vivo or by its derivatives that are produced by leukocytes during inflammatory processes. These data further emphasize the conservative aspect of MATS-PBT analysis potentially leading to an underestimation of strain coverage. The de-repression of *nadA* is expected to lead to higher levels of NadA expression from *nadA*-positive strains and to increased killing by anti-NadA antibodies elicited by the 4CMenB vaccine [38]. Of note, PorA P1.4 was predicted to cover not only 50% of isolates belonging to cc41/44, a clonal complex which usually associated with PorA VR2 4, but also 3% of isolates belonging to cc162.

Recently, five European meningococcal reference laboratories were involved in a MATS standardization study (Euro-5, comprising Germany, France, Italy, the United Kingdom and Norway) [23] with an addition of Czech Republic and Spain providing their estimates. Beyond this first European study, there is a need for further investigations of strain coverage by clonal complex since the clonal complex distribution may vary on a country-by-country basis and the predicted strain coverage might be consequently different. The present study provides additional evidence on the predicted coverage for meningococci B cc162 that in a previous European study were less representative.

The coverage predicted by MATS-PBT for the 52 strains collected in Greece during 2008–2010, a time frame comparable with the period considered by the Euro-5 study, was 88%. This estimation fell in the range of coverage observed among the Euro-5 countries regardless of the geographical distribution of the clonal complexes. For instance, despite the prevalence of cc162 in the total 148 isolates, the most prevalent cc in Greece among the 52 isolates from 2008 to 2010, was cc269 (44.2%), which was well covered (97%) by 4CMenB. cc269 accounted for 19.5% in the Euro-5 study and was absent in Italy. The overall frequency of coverage by at least two antigens was similar (44.6% vs. 49.8%) in Greek and in Euro-5 isolates respectively and the Greek isolates fell within the range observed among the five countries (39.6% for Italy to 53.9% for Germany) [23]. Since lack of coverage due to point mutations is less likely for strains expressing multiple vaccine antigens, the percentage of Greek strains covered by at least two vaccine antigens suggests that the rate of emergence of escape variants in Greece is not expected to be different than in other European countries. More recently, a study on estimate of 4CMenB coverage of 157 Canadian serogroup B isolates circulating from 2006 to 2009 has also been published [24] In Canada, where the most frequent ccs were cc41/44 and cc269, the overall 4CMenB MATS predicted coverage was 66%, slighly lower than in Greek and Euro-5 isolates, however results were similar to those found in England and Wales.

## Conclusions

At present, there is an increasing number of reports published using MATS. Nevertheless, there has been, up to now, no data from Greece. Our data provide a good prediction of the potential coverage of 4CMenB in Greece similarly to other European countries, despite differences in the prevalence of MLST genotypes, such as cc162 and, as a consequence, in the frequency and distribution of fHbp, NHBA and NadA protein peptides. However, our study argues for continuous surveillance by MATS typing that should allow “real-time” post-implementation estimates of coverage.

## Authors’ contributions

GT, MT, MP participated in the study design and the preparation of the manuscript, EH, KK, AX participated in the laboratory experimental work and in the interpretation of data, SB, AM, LO and MC participated in the analysis of the data.

## Authors’ information

*GT* PhD, Head, National Meningitis Reference Laboratory, National School of Public Health Athens, Greece. *EH* BSc Institute Pasteur, Invasive Bacterial Infections Unit, Paris, France. KK PhD National Meningitis Reference Laboratory, National School of Public Health Athens, Greece. *AX* PhD National Meningitis Reference Laboratory, National School of Public Health Athens, Greece. SB PhD Novartis Vaccines and Diagnostics, Siena, Italy. LO Msc Novartis Vaccines and Diagnostics, Siena, Italy. MC PhD Novartis Vaccines and Diagnostics, Siena, Italy. AM PhD Novartis Vaccines and Diagnostics, Siena, Italy. M-KT MD, PhD Institute Pasteur, Head, Invasive Bacterial Infections Unit, Paris, France.
